# Clinically Relevant Changes for Cognitive Outcomes in Preclinical and Prodromal Cognitive Stages: Implications for Clinical Alzheimer Trials

**DOI:** 10.1212/WNL.0000000000200817

**Published:** 2022-09-13

**Authors:** Emma Borland, Chris Edgar, Erik Stomrud, Nicholas Cullen, Oskar Hansson, Sebastian Palmqvist

**Affiliations:** From the Clinical Memory Research Unit (E.B., E.S., N.C., O.H., S.P.), Department of Clinical Sciences, Lund University; Department of Neurology(E.B.), Skåne University Hospital, Malmö, Sweden; Department of Clinical Science (C.E.), Cogstate, London, United Kingdom; and Memory Clinic (E.S., O.H., S.P.), Skåne University Hospital, Malmö, Sweden.

## Abstract

**Background and Objectives:**

Identifying a clinically meaningful change in cognitive test score is essential when using cognition as an outcome in clinical trials. This is especially relevant because clinical trials increasingly feature novel composites of cognitive tests. Our primary objective was to establish minimal clinically important differences (MCIDs) for commonly used cognitive tests, using anchor-based and distribution-based methods, and our secondary objective was to investigate a composite cognitive measure that best predicts a minimal change in the Clinical Dementia Rating—Sum of Boxes (CDR-SB).

**Methods:**

From the Swedish BioFINDER cohort study, we consecutively included cognitively unimpaired (CU) individuals with and without subjective or mild cognitive impairment (MCI). We calculated MCIDs associated with a change of ≥0.5 or ≥1.0 on CDR-SB for Mini-Mental State Examination (MMSE), ADAS-Cog delayed recall 10-word list, Stroop, Letter S Fluency, Animal Fluency, Symbol Digit Modalities Test (SDMT) and Trailmaking Test (TMT) A and B, and triangulated MCIDs for clinical use for CU, MCI, and amyloid-positive CU participants. For investigating cognitive measures that best predict a change in CDR-SB of ≥0.5 or ≥1.0 point, we conducted receiver operating characteristic analyses.

**Results:**

Our study included 451 cognitively unimpaired individuals, 90 with subjective cognitive decline and 361 without symptoms of cognitive decline (pooled mean follow-up time 32.4 months, SD 26.8, range 12–96 months), and 292 people with MCI (pooled mean follow-up time 19.2 months, SD 19.0, range 12–72 months). We identified potential triangulated MCIDs (cognitively unimpaired; MCI) on a range of cognitive test outcomes: MMSE −1.5, −1.7; ADAS delayed recall 1.4, 1.1; Stroop 5.5, 9.3; Animal Fluency: −2.8, −2.9; Letter S Fluency −2.9, −1.8; SDMT: -3.5, −3.8; TMT A 11.7, 13.0; and TMT B 24.4, 20.1. For amyloid-positive CU, we found the best predicting composite cognitive measure included gender and changes in ADAS delayed recall, MMSE, SDMT, and TMT B. This produced an AUC of 0.87 (95% CI 0.79–0.94, sensitivity 75%, specificity 88%).

**Discussion:**

Our MCIDs may be applied in clinical practice or clinical trials for identifying whether a clinically relevant change has occurred. The composite measure can be useful as a clinically relevant cognitive test outcome in preclinical AD trials.

The minimal clinically important difference (MCID) is defined as the smallest change on a measure that is reliably associated with a meaningful change in a patient's clinical status, function, or quality of life.^1^ It is important to decide the smallest change in an outcome that constitutes a clinically meaningful change—that is, MCID—to interpret whether, for example, the treatment effect measured using a cognitive test is clinically relevant or whether a change in cognitive testing during a clinical follow-up represents a clinically meaningful change in cognition. MCIDs are thus necessary to make accurate clinical decisions and to design clinical trials with the statistical power to detect an effect equal to or greater than the MCID.^2^

In the 2018 US Food and Drug Administration (FDA) guidance for clinical trials in early AD, the guidance introduced a clinical staging framework for AD stages 1–3.^3^ Stage 1 includes individuals with abnormal biomarkers without cognitive complaints or detectable decline even on sensitive tests. Stage 2 includes individuals with subtle cognitive effects without functional deficits, and stage 3 includes individuals beginning to have difficulties with daily tasks. To presume a drug has a clinically beneficial effect for individuals in stage 2, the agency states that a pattern of beneficial effects on neuropsychological assessments is more persuasive if seen on multiple tests and that if only seen on 1 assessment, it needs to show a large magnitude of effect to be persuasive of a beneficial effect. However, for many cognitive assessments, the magnitude that corresponds to a clinically meaningful effect compared with that for placebo is unknown.

Several methods exist for estimating a meaningful clinical effect, among which the most well-established are anchor-based and distribution-based estimates.^4^ Anchor-based approaches to determine meaningful within-patient change involve the use of an external reference with an already established relevance.^5^ Distribution-based, or internal estimates, use statistical properties of the measures themselves, and of them, the most common are effect size metrics—for example, the SD and the SEM that incorporate some measure of scale reliability (e.g., test-retest or Cronbach α as a measure of internal consistency reliability).

In addition to establishing clinically relevant cutoffs for test changes, it is also important to determine which tests best represent clinically relevant changes. The preclinical Alzheimer cognitive composite (PACC) has earlier been proposed as an outcome measure sensitive for early cognitive changes in AD (stages 1 and 2).^6^ The PACC was initially created by selecting 4 well-established cognitive tests that are sensitive to detecting change/worsening in prodromal and mild dementia and with sufficient range to also detect early decline in preclinical stages of disease.^6^ However, the PACC was established purely based on the presumed sensitivity to detect changes and not whether the changes were clinically meaningful. We propose that by developing and validating cognitive composites and test batteries using predictive validity for a clinically important change incorporating anchor-based approaches, more relevant outcomes may be developed than by focusing on within-measure change/worsening, which is distribution-based alone.

The aims of this study were (1) to establish cutoffs for cognitive test changes for use to conclude whether a meaningful magnitude of treatment effect has been achieved and (2) to investigate which single and combinations of cognitive test differences best corresponds to a clinically meaningful decline. In addition to examining the second aim in cognitively unimpaired (CU) participants and participants with mild cognitive impairment (MCI), we also examined this in Aβ-positive CU participants because this is a group of special interest in present and future clinical AD trials.^7^

## Methods

### Population

The participants in the study were consecutively included from the prospective Swedish BioFINDER study (biofinder.se), and participants for this study were enrolled from July 6, 2009, to March 4, 2015. The population consisted of 451 CU individuals and 292 people classified as experiencing mild cognitive impairment (MCI). In the CU group, 90 individuals experienced subjective cognitive decline, and 361 people were cognitively healthy controls based on a structured assessment. The individuals were followed up longitudinally (for CU pooled mean for all different tests 32.4 months (pooled SD 26.8, range 12–96 months), MCI pooled mean 19.2 months (pooled SD 19.0, range 12–72 months), with a mean number of data points of 1588 for CU and 727 for MCI.

MCI was defined according to the performance on a comprehensive neuropsychological battery, as previously described.^8^ All cognitively unimpaired indviduals had a Clinical Dementia Rating—Sum of Boxes at inclusion of 0. Participants with MCI were excluded after converting to major neurocognitive disorder. Participants were assessed by physicians well experienced in dementia disorders, underwent a physical examination, MRI scan, lumbar punction, and cognitive assessments, and were rated with the CDR. Participants experiencing cognitive symptoms at baseline (subjective cognitive disease or MCI) were followed up annually, while participants without cognitive symptoms at baseline were examined every second year by physicians.

### Cognitive Tests

Eight cognitive tests were examined in this study, covering the cognitive domains of executive function, attention, episodic and semantic memory, and visuospatial function. Participants were examined with the Mini-Mental State Examination (MMSE), the Alzheimer Disease Assessment Scale (ADAS) 10-word delayed recall, Letter S Fluency, Animal Fluency, Stroop Color and Word Test (Stroop), Trailmaking Tests A and B, and Symbol Digit Modalities Test. Further explanation of tests, what they assess, and how points are counted are described in eMethods.

### Clinical Dementia Rating

Clinical Dementia Rating (CDR) is an ordinal scale with scores of 0–3 points used to quantify the functional effect of cognitive impairment (0 = none, 0.5 = questionable, 1 = mild, 2 = moderate, and 3 = severe) in domains (box scores) of memory, orientation, judgment and problem solving, community affairs, home and hobbies, and personal care.^9,10^ Participants in this study were rated on the CDR by a thorough review of patient records where dementia experts assessed cognitive symptoms and activities of daily living (ADL) in comprehensive semistructured interviews with patients and informants, including informant-based questionnaires of ADL (the functional activities questionnaire)^11^ and cognitive symptoms (the CIMP-QUEST)^12^ (supported if necessary by cognitive test results). The CDR scale provides a quantitative index of impairment, referred to as the Sum of Boxes or CDR-SB (range of 0–18), and may also be scored in the CDR global severity stage score (range 0–3) using an algorithm. An increase in the CDR-SB score has been identified as having both face and predictive validity to identify people who are later diagnosed with probable AD or another dementia.^13^ For predementia AD, a single box score increment of either 0.5 or 1.0 has been proposed to capture efficacy and clinical relevance for early AD.^14^ A change of 1 in CDR-SB could be either a change of 0.5 points in 2 boxes or a change of 1 point in 1 box.

### Determining Amyloid Positivity

The procedure and analysis of CSF followed the Alzheimer Association Flowchart for CSF biomarkers.^15,16^ We used the ratio for Aβ42/40 that we establish acquire through CSF analysis. CSF Aβ42 and Aβ40 were analyzed using the Roche Elecsys CSF immunoassays (NeuroToolKit) on all participants. The cutoff for Aβ42/40 was established with mixture modeling statistics^17,18^ and set at 0.066.

### Statistical Analysis

The psychometric criterion reliable change index (RCI) is used to evaluate whether a change over time of an individual score is considered statistically significant.^19^ RCI provides a CI that represents the predicted changes that would occur if a patient's test score does not change significantly from one assessment to another. We calculated the RCI for all 8 test differences with a 90% CI (the most common CI for an RCI)^19^ for CU participants and participants with MCI. This is performed for tests separately using the following equation:

            
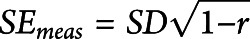


            
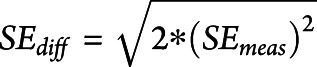


            
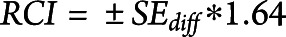


 SD = Standard deviation of the test score at baseline

 r = Pearson coefficient for test results in cognitively stable individuals

 SE_meas_ = standard error of measurement

 SE_diff_ = standard error of differentiation

Estimates of effect size (ES) are useful for determining the magnitude or size of an effect, the relative contribution of different factors or the same factor in different circumstances, and the power of an analysis.^20^ ES is defined as a mean difference in score divided by standard deviation of baseline scores. An ES of 0.5 is generally considered a moderate clinically significant change, whereas an ES of 0.2 is considered a small change and 0.8 a large change.^21,22^ The standardized response mean (SRM) is an effect size used to measure the responsiveness of outcome measures (the ability to detect change over time), defined as mean difference in score divided by SD of the change from previous visit score.^23^ ES and SRM were calculated for all test changes in CU and MCI participants. Experts have previously defined a clinically meaningful cognitive decline as a decline in cognitive function of 0.5 or more SDs from baseline cognitive scores,^24-26^ which we present in our results.

For the anchor-based approach, we analyzed mean differences in cognitive test scores anchored to differences in CDR-Sum of Boxes (CDR-SB) scores. For the CU individuals, we used a change of CDR-SB ≥0.5 points and for the MCI group a change of ≥1 point as anchors to represent a clinically meaningful change. We calculated the mean, SD, and ES for changes in the cognitive tests separately for meaningful decline (CDR-SB difference of ≥0.5 and ≥1) and no meaningful decline (CDR-SB difference of 0).

According to previously described methods, MCIDs are recommended to be triangulated (calculated of the arithmetic mean) to produce a final MCID for each mean value.^27^ Triangulation integrates results from ratings with clinical changes, statistical estimates, and qualitative data from patients and/or clinicians to derive guidelines.^28^ A previously suggested method is to assign the anchor-based results a weight of two-thirds and the distribution-based method a weight of one-third.^29^ The final triangulated MCID is then calculated as the mean values of these 3 parts. Our anchor-based MCIDs are estimated from ES (based on clinical changes measured with CDR), and distribution-based MCIDs are based on statistical measures (SEM). Because an ES of 0.5 is generally considered a clinically significant change, we used the estimated anchor-based MCID with the ES closest to 0.5.

To examine which tests that best represented a clinically meaningful change, we analyzed the cognitive tests as independent variables in logistic regression models with CDR-SB as a dependent variable. For the CU group, the CDR-SB difference was dichotomized as either 0 (no clinical change) or ≥0.5 point change (smallest clinically relevant change). For the MCI group, we dichotomized with a larger CDR-SB difference as either 0 or ≥1 point; this excluded between 55 and 159 data points depending on the test because of a CDR-SB difference of 0.5. The area under ROC (AUC) curve and sensitivity and specificity for each test difference were calculated using ROC analyses. Logistic regression models were performed on a subsample with complete data for the analyzed cognitive tests (i.e., all logistic regression models were performed on the same population). To find the most optimal combination of test differences to estimate a cognitive change, we examined all cognitive test changes in the model for CU to identify a model with the lowest Akaike Information Criterion (AIC). AIC accounts for the trade-off between model fit and sparsity (as few included biomarkers as possible) to protect against model overfitting and can be used as a tool for model selection.^30,31^ Lower AIC indicates a better model. To find the optimal combination for MCI, we excluded Animal Fluency, Letter S, and TMT B because these tests were only conducted every second year and therefore excluded many participants because of lack of complete cases. We added age at visit, education years, and gender in the models. Predictors with a *p* value >0.1 were removed from the model.

### Standard Protocol Approvals, Registrations, and Patient Consents

The study was approved by the regional ethical committee at Lund University, Lund, Sweden. All participants gave their written informed consent to participate in the study.

### Data Availability

Anonymized data will be shared upon request from a qualified academic investigator for the sole purpose of replicating procedures and results presented in the article and as long as data transfer is in agreement with the European Union legislation on the general data protection regulation and decisions by the Swedish Ethical Review Authority and Region Skåne, which should be regulated in a material transfer agreement.

## Results

Baseline characteristics are summarized in Table 1. Four hundred fifty-one people were included in the cognitively unimpaired (CU) group (mean number of data points for each test 1,588) and 292 people in the MCI group (mean number of data points 727). The mean (SD) age of the CU group was 72.9 (5.5) years and that of the MCI group 71.1 (5.5) years. 116 (16%) people in the CU group were amyloid positive. Pooled mean time between test results for CU participants was 32.4 months (pooled SD 26.8), for MCI 19.2 months (pooled SD 19.0), and for CU amyloid positive 32.5 months (pooled SD 27.2).

**Table 1:**
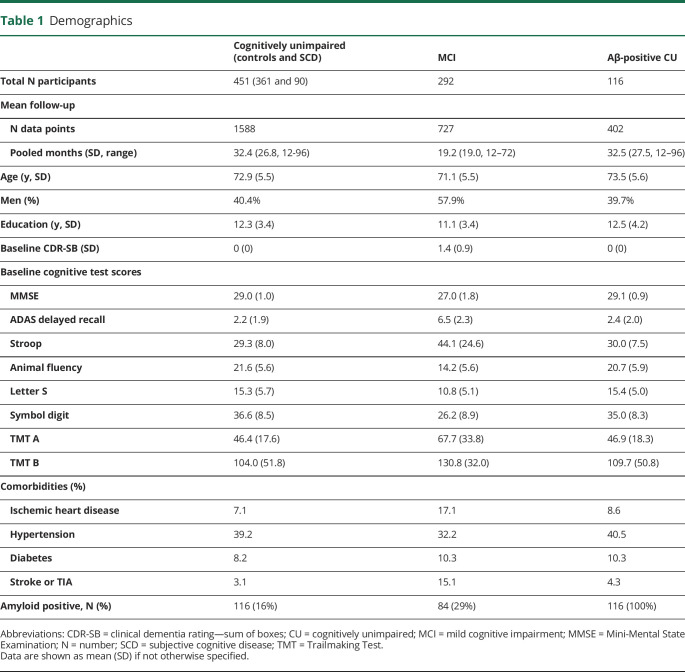
Demographics

Table 2 summarizes the RCIs, that is, the 90% CIs where a change above that interval indicates a significant change in the test. In summary, for MMSE, ADAS, and Animal Fluency, we found similar RCIs for the CU and MCI groups, whereas in Stroop, Letter S, Symbol Digit, TMT A and B, we found larger RCIs for the MCI group than the CU group.

**Table 2:**
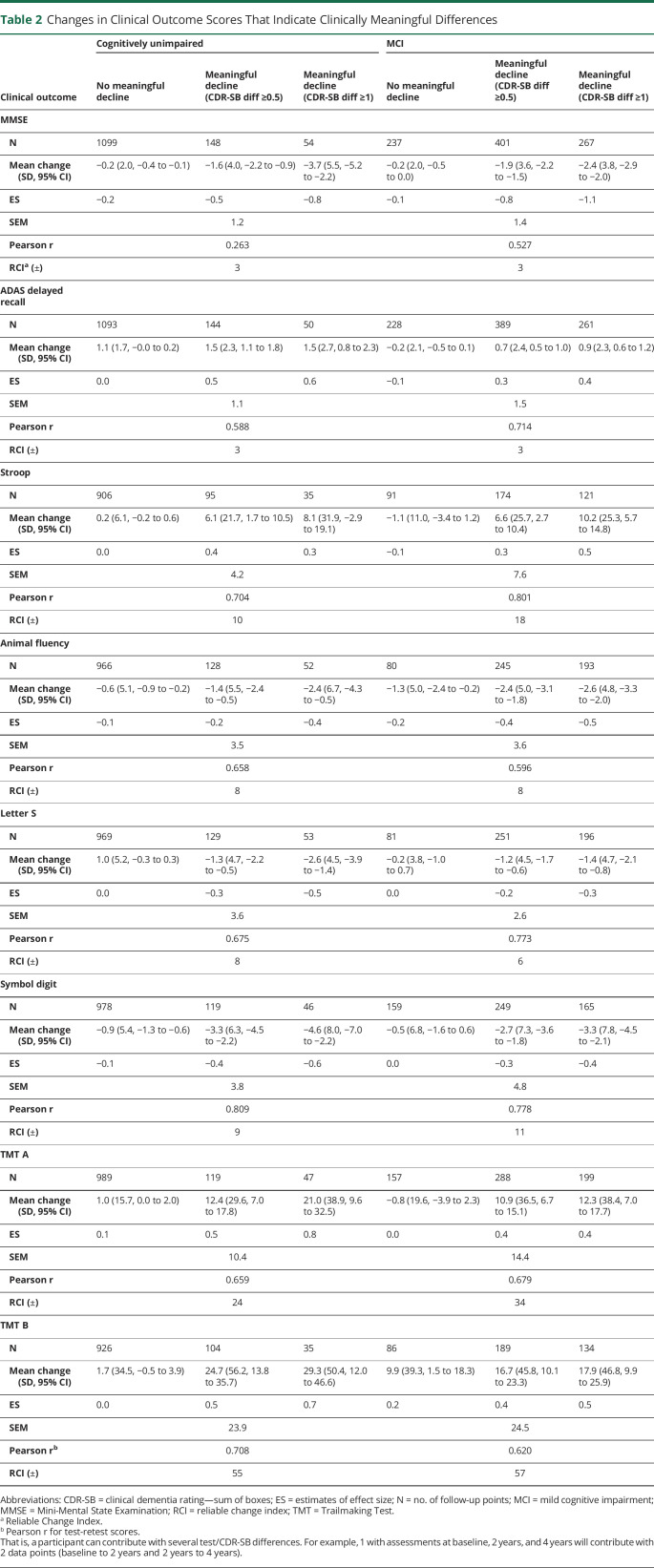
Changes in Clinical Outcome Scores That Indicate Clinically Meaningful Differences

**Figure 1:**
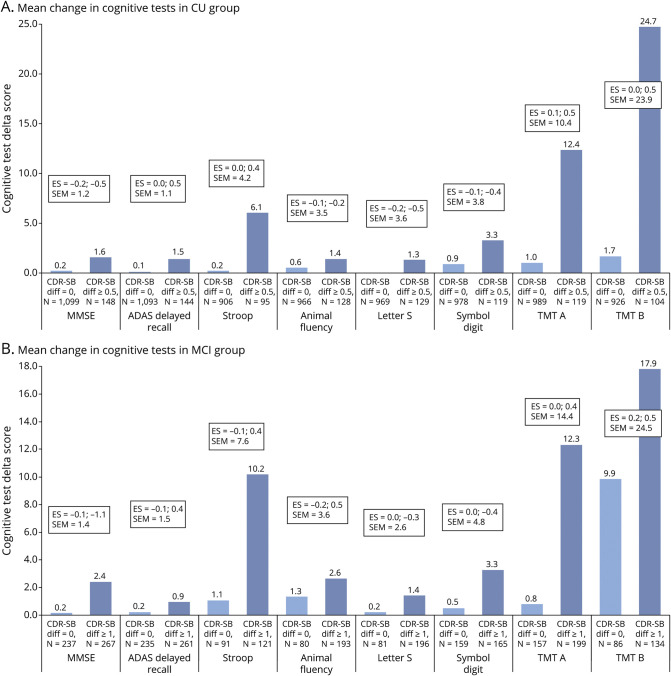
Changes in Clinical Outcome Scores That Indicate Clinically Meaningful Differences (A) Cognitively unimpaired, (B) Mild Cognitive impairment. For MMSE, Animal Fluency, Letter S and Symbol Digit all mean difference scores are negative (see Table 2). ES = estimates of effect size.

Correlation coefficients between the change in test score and change in CDR-Sum of Boxes (CDR-SB) are summarized in eTable 1 (links.lww.com/WNL/C168). We found significant correlations between CDR-SB and all cognitive tests, however weak to moderate (correlation coefficients −0.2–0.6 depending on test). We calculated triangulated MCIDs for each test to derive cutoffs for cognitive test differences that are both empirically and clinically relevant for deciding whether a change in each test represents a meaningful change. We listed estimated MCIDs for group-based clinical worsening or progression in CU and MCI participants using the anchor-based (mean change [SD], ES, and SEM) and distribution-based methods (Pearson for test-retest and RCI) in Table 2 and Figure 1 and triangulated MCIDs for CU and MCI participants in Table 3. Supplementary data for MCIDs (1/2 SD of baseline and SRM) are summarized in eTable 2 (links.lww.com/WNL/C168).

**Table 3:**
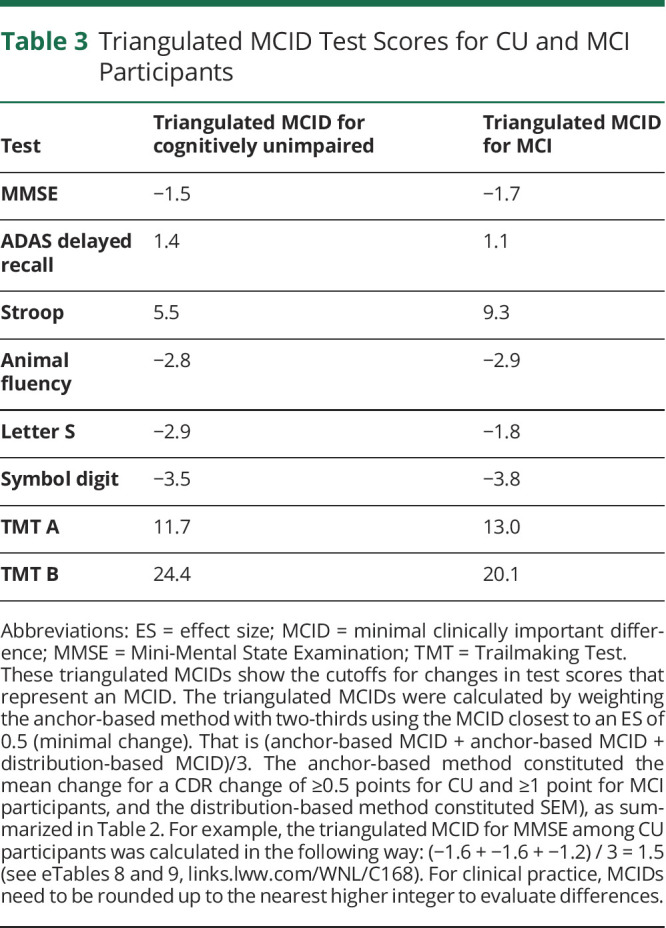
Triangulated MCID Test Scores for CU and MCI Participants

**Figure 2:**
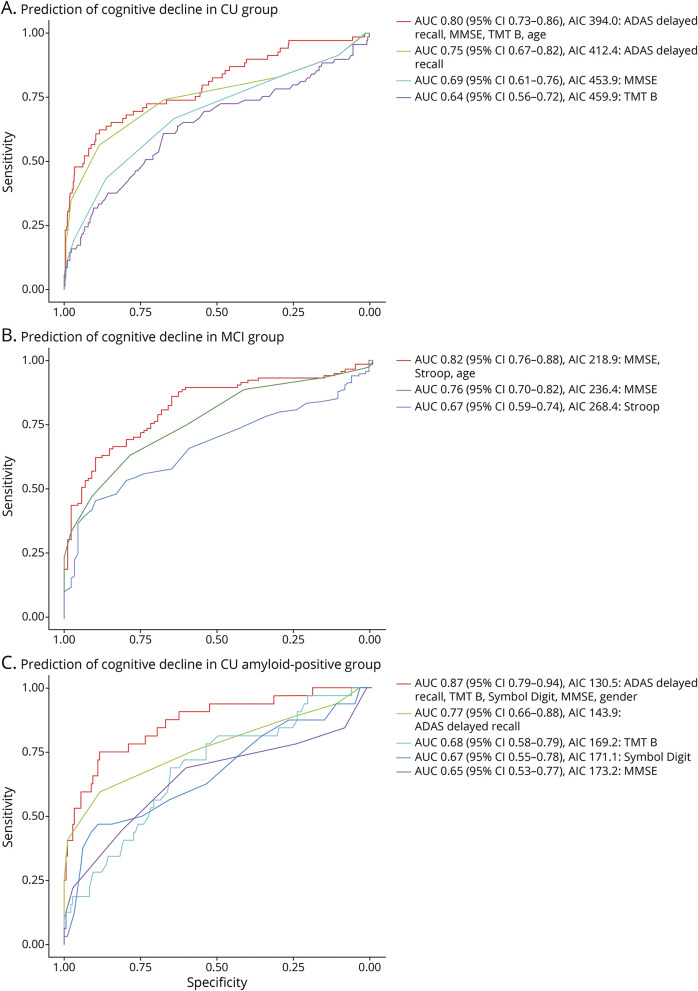
ROC Curves From Logistic Regression Models Estimating Minimally Clinical Difference in CU, MCI, and Amyloid positive CU groups. (A) CU group (N=451). (B) MCI group (N=292). (C) Amyloid positive CU group (N=116). Performance of the best model (defined as lowest AIC including predictors with *p* < 0.1) and individual tests are shown in receiver operating curves (ROC). Further results are presented in eTables 3-6. Outcomes in A and C are a clinical change in Clinical Dementia Rating Sum of Boxes (CDR-SB) ≥0.5 and in C a clinical change in CDR-SB ≥1 point.

Next, we examined how accurately differences in test scores could estimate a minimal clinically relevant change using logistic regression models (see Figure 2 and eTable 3, links.lww.com/WNL/C168). A clinical change was defined as discrimination between the progression of CDR-SB of ≥0.5 vs CDR SB change of 0 for CU and 0 vs ≥1 for MCI participants. For CU (amyloid-positive and amyloid-negative) partcipants, the best predicting univariate test was ADAS delayed recall (AUC 0.75) and the worst Animal Fluency (AUC 0.54). For MCI paticipants, MMSE was the best predicting test (AUC 0.75) and TMT A the least (AUC 0.61), taken in account that Animal Fluency, Letter S, and TMT B were excluded from the group because of lack of follow-up data. Univariate analyses are summarized in eTables 4–6 (links.lww.com/WNL/C168). When combining the tests, we found that the best model (the lowest AIC) for CU individuals was a combination of age and changes in ADAS delayed recall, MMSE, and TMT B, which produced an AUC of 0.79 (95% CI 0.72–0.86) for identifying a clinical change (discrimination between the progression of CDR-SB of ≥0.5 vs CDR SB change of 0). For MCI participants, the best predicting model included changes in Stroop, MMSE, and age, which had an AUC of 0.82 (95% CI 0.76–0.88). For the amyloid-positive CU group, we found the best predicting composite cognitive measure included changes in ADAS delayed recall, Stroop, Symbol Digit, and TMT B, including gender in the model. We performed a stepwise backward selection, removing the least significant variables, starting with Stroop because this was not significant (*p* = 0.12). After removing Stroop, we found the best predicting composite cognitive measure included ADAS delayed recall, MMSE, symbol digit modalities test, TMT B, and gender. This produced an AIC of 130.5 and AUC of 0.87 (95% CI 0.79–0.94, sensitivity 75%, specificity 88%).

## Discussion

We have established minimally clinically important differences (MCIDs) for group-based worsening in test scores for 8 commonly used cognitive tests to help guide clinicians and researchers on clinically relevant cognitive decline with repeated assessments. We investigated changes in cognitive tests longitudinally with distribution-based and anchor-based methods. The distribution-based MCIDs were generally higher (i.e., a larger test change required to indicate an MCID) than the anchor-based MCIDs, showing the importance of using clinical measures of importance (such as CDR) according to the population. We found the best predicting model for a clinical change included differences in test results in ADAS delayed recall, MMSE, and TMT B for cognitively unimpaired (CU), Stroop, MMSE, and age for MCI, and ADAS delayed recall, MMSE, symbol digit modalities test, TMT B, and gender for amyloid-positive CU individuals.

The novelty of this study is that we present MCIDs for several cognitive tests that, to our knowledge, has not been studied before, which could be used in future clinical AD trials for establishing clinical meaningful treatment effect for treatments seeking to prevent or slow disease progression. Besides, this study has the advantage of presenting triangulated data for MCIDs representing clinical changes, statistical estimates, and qualitative data from clinicians using CDR ratings. When triangulating MCIDs, we integrate results from ratings of clinical changes from ES (based on clinical changes measured with CDR) and statistical estimates (SEM). To our knowledge, previous studies have not investigated which tests best predict a cognitive decline using anchor-based methods, and in this study, we present this for CU individuals, individuals with MCI, and specifically amyloid-positive CU participants, which is the target population of several large ongoing AD trials.

The present findings are important because there is no previous consensus on MCIDs for cognitive test outcomes in AD trials; yet FDA specifically highlights that a clinically meaningful improvement on cognitive test scores should be shown before approval of the drug.^3^ Recent trials on treatment for AD have investigated changes in cognition comparing individuals receiving placebo with those under active treatment. In the EMERGE study,^2^ the population receiving high-dose treatment with the antiamyloid treatment aducanumab reported a statistically significant reduced decline of 0.6 points on the MMSE between placebo and aducanumab groups favoring aducanumab. However, using our MMSE MCID (1.7 points) would render this mean change clinically insignificant. In the TRAILBLAZER-ALZ^2^ study for Donanemab including individuals with MCI-mild AD, they found a difference on MMSE of 0.64 between the placebo and Donanemab cohorts, which again would not be clinically significant.

Previous studies have shown similar or larger MCIDs for MMSE compared with our results between 1 and 4 points;^32-35^ however, we have not found previous estimated MCIDs for the other examined tests. One previous study on MCID for MMSE suggested a 0.4 SD change from baseline for MMSE as MCID, corresponding to an MCID of 1.4 MMSE points,^33^ close to results from another study showing an MCID for MMSE of 1.6 points for 0.4 SD from baseline MMSE,^34^ close to our calculated MCIDs (−1.5 MMSE points for the CU group and −1.7 for the MCI group). Another previous study showed an estimated MCID for MMSE of 1–3 points depending on disease severity, with larger results using only distribution-based approach similar to our study.^32^ Yet another study has showed a far higher MCID of MMSE of 3.72 points.^35^ We found a very low 0.5 SD of baseline MMSE (1.1 for CDR SB change of ≥0.5 in CU), which is partly caused by the inclusion criteria in the BioFINDER study for the CU group of MMSE score ≥28 points but does not explain why the MCI group had the same results. The estimated RCIs are much larger than MCID explained by the methodology with a large SD (1.65), being individual patient–based, differing from MCID as being minimal change at the group level. Much smaller changes may in fact be relevant as seen in our calculated MCIDs.

The CDR global has been used as an external anchor to establish meaningful change estimates for other scales.^36^ While it has clinical validity as a meaningful change, progression from one stage to another represents a change that is much larger than what may be considered minimally important, which is why we have chosen to use the CDR-Sum of Boxes (CDR-SB) as an anchor for this study. Previous studies have also shown that to identify MCI, CDR-SB might be more accurate than global CDR.^37^ Studies have reported a high internal consistency for the CDR-SB across the AD spectrum with a low variability in mean changes^38^ and that mean scores decline nearly linearly.^39^ In summary, we therefore chose to use CDR-SB as the anchor for determining clinically meaningful important differences in cognitive test results. In a recent study, it was shown that CDR-SB was not strongly correlated with the cognitive assessments MMSE or ADAS-Cog at baseline; however, there was a moderate correlation between change in CDR-SB and ADAS-Cog13 (r = 0.5) and MMSE (r = −0.4) at a 2-year follow-up. The same study showed that both CDR-SB and MMSE had a strong responsiveness to change.^10^ In our study, we have seen significant correlations between CDR-SB and all cognitive tests, however weak to moderate (correlation coefficients −0.17–0.63 depending on test, see eTable 1, links.lww.com/WNL/C168). Previous studies have recommended a correlation of at least 0.3–0.35 between the change score and the anchor.^40^ In our study, Animal Fluency (CU only), Letter S (CU and MCI), and TMT B (MCI only) had correlations <0.3 with CDR-SB as the anchor.

To our knowledge, no previous study has investigated which combination of cognitive tests best estimate clinically relevant yet minimal worsening in CDR-SB. The most frequently used cognitive composite is the PACC, which originally included the Free and Cued Detective Reminding Test, logical memory, Digit Symbol Substitution Test (equivalent to the symbol digit test used in this study), and the MMSE.^6^ Later, modified PACC versions have included animal fluency, TMT B, symbol digit, and/or ADAS delayed recall.^41,42^ Using our model selection approach, we could confirm that a combination of TMT B, ADAS delayed recall, Symbol Digit, and MMSE indeed not only are sensitive to cognitive changes over time as shown previously but also represent a clinically meaningful change. We did not find that changes in Animal Fluency were accurate in estimating a minimal meaningful decline. Overall, we found the best combined model of changes in cognitive tests with logistic regression models and found for amyloid-positive CU individuals the best model combined differences in cognitive test results in ADAS delayed recall, MMSE, TMT B, and Symbol digit combined with patient's gender, which includes all 3 cognitive domains.^6^ We suggest that this technique could be used to develop other clinically relevant cognitive composites and test batteries for use in predementia populations, using broader cognitive test batteries to find the best model for predicting a cognitive change.

A potential limitation to the study is that the follow-up of participants is annual for MCI participants and every second year for most CU participants (annual for those with subjective cognitive symptoms at baseline), which might result in missing some fluctuation or decline in cognition in CU individuals. However, because the primary approach is based on an anchor, this should not largely affect MCID estimates. In addition, any progression occurs slower and less frequent in CU participants, which is why the study was designed to have less frequent follow-ups for controls. Another limitation is that MCI participants can potentially fluctuate and revert to CU. Unfortunately, we have not classified participants as CU or MCI at follow-ups; however, if using a CDR-SB of 0 points as a proxy for being CU, only 2.0%–4.7% reverted from CDR-SB>0 to 0 in the MCI group (eTable 7, links.lww.com/WNL/C168), why it should have very little relevance to the results. Furthermore, the purpose of the present estimates is to elucidate what may be a meaningful change in cognitive and clinical status; irrespective of whether such changes remain stable or represent continuous progression of underlying disease, they are still relevant to how people feel and function. A limitation to our calculated MCIDs is that Stroop violates the expectation of ordered ES (partly due to lower N [N = 35] and an increase in SD), and it should therefore be interpreted with caution. This sample-dependent nature is a challenge to the use of ES in general. An alternative would have been to use a CDR-SB difference of ≥0.5 in all cases as minimal and defined as the smallest difference a clinician is able to observe and score; however, we reason that the magnitude of the change in score could then potentially be too small to be clinically meaningful. In general, this is why we seek to use both anchor and distribution in generating estimates and not just the latter and give priority to the anchor.

Our triangulated MCIDs for cognitive test measures could potentially be applied in clinical practice to evaluate whether a clinical progression has occurred since last visit or whether the patient has remained stable. However, further work would be needed to define cutoffs representing possible scores on the instruments, as opposed to aggregate, group-level changes. The results from the logistic regression models (Figure 2 and eTable 3, links.lww.com/WNL/C168) suggests the suitable tests depending on setting (CU, MCI, or amyloid-positive CU) and Table 3 cutoffs that indicate that a meaningful change in the test has occurred. However, in clinical practice, MCIDs need to be rounded up to the nearest higher integer to evaluate differences. This selection of tests and identified cutoffs should however be validated in independent and more diverse populations with wider age range and education level. The MCIDs can also help to identify treatment benefits in clinical trials of therapies for early AD, and as we have reported earlier, several new studies on pharmaceutical treatments for AD have found significant changes in cognitive outcomes but may not be clinically relevant.

## Glossary

AD: Alzheimer Disease
ADAS: Alzheimer's Disease Assessment Scale
ADL: activities of daily living
AIC: Akaike Information Criterion
AUC: area under the ROC curve
CDR-SB: clinical dementia rating—sum of boxes
CU: cognitively unimpaired
ES: estimates of effect size
FDA: Food and Drug Administration
MCI: mild cognitive impairment
MCID: minimal clinically important difference
MMSE: Mini-Mental State Examination
PACC: preclinical Alzheimer cognitive composite
RCI: reliable change index
SDMT: Symbol Digit Modalities Test
SRM: standardized response mean
TMT: Trailmaking Test

## References

[1] Malec JF, Kean J, Monahan PO. The minimal clinically important difference for the Mayo-Portland adaptability inventory. J Head Trauma Rehabil. 2017;32(4):E47-E54. 28489702 10.1097/HTR.0000000000000268 PMC5432408

[2] Liu KY, Schneider LS, Howard R. The need to show minimum clinically important differences in Alzheimer's disease trials. Lancet Psychiatry. 2021;8(11):1013-1016. 34087114 10.1016/S2215-0366(21)00197-8

[3] FDA. Early Alzheimer's disease: developing drugs for treatment guidance for industry. 10.1016/j.trci.2018.11.004 PMC6804505 31650002

[4] Edgar CJ, Vradenburg G, Hassenstab J. The 2018 revised FDA guidance for early alzheimer's disease: establishing the Meaningfulness of treatment effects. J Prev Alzheimers Dis. 2019;6(4):223-227. 31686092 10.14283/jpad.2019.30

[5] Phillips GA, Wyrwich KW, Guo S, et al. Responder definition of the Multiple Sclerosis Impact Scale physical impact subscale for patients with physical worsening. Mult Scler. 2014;20(13):1753-1760. 24740371 10.1177/1352458514530489 PMC4232315

[6] Donohue MC, Sperling RA, Salmon DP, et al; Australian Imaging, Biomarkers, and Lifestyle Flagship Study of Ageing; Alzheimer’s Disease Neuroimaging Initiative; Alzheimer’s Disease Cooperative Study. The preclinical Alzheimer cognitive composite: measuring amyloid-related decline. JAMA Neurol. 2014;71(8):961-970. 24886908 10.1001/jamaneurol.2014.803 PMC4439182

[7] Sperling RA, Rentz DM, Johnson KA, et al. The A4 study: stopping AD before symptoms begin? Sci Transl Med. 2014;6(228):228fs13. 10.1126/scitranslmed.3007941 PMC4049292 24648338

[8] Petrazzuoli F, Vestberg S, Midlov P, Thulesius H, Stomrud E, Palmqvist S. Brief cognitive tests used in primary care cannot accurately differentiate mild cognitive impairment from subjective cognitive decline. J Alzheimers Dis. 2020;75(4):1191-1201. 32417771 10.3233/JAD-191191 PMC7369041

[9] Hughes CP, Berg L, Danziger WL, Coben LA, Martin RL. A new clinical scale for the staging of dementia. Br J Psychiatry. 1982;140:566-572. 7104545 10.1192/bjp.140.6.566

[10] McDougall F, Edgar C, Mertes M, et al. Psychometric properties of the clinical dementia rating - Sum of boxes and other cognitive and functional outcomes in a prodromal alzheimer's disease population. J Prev Alzheimers Dis. 2021;8(2):151-160. 33569561 10.14283/jpad.2020.73

[11] Pfeffer RI, Kurosaki TT, Harrah CH Jr, Chance JM, Filos S. Measurement of functional activities in older adults in the community. J Gerontol. 1982;37(3):323-329. 7069156 10.1093/geronj/37.3.323

[12] Astrand R, Rolstad S, Wallin A. Cognitive Impairment Questionnaire (CIMP-QUEST): reported topographic symptoms in MCI and dementia. Acta Neurol Scand. 2010;121(6):384-391. 20055769 10.1111/j.1600-0404.2009.01312.x

[13] Dickerson BC, Sperling RA, Hyman BT, Albert MS, Blacker D. Clinical prediction of Alzheimer disease dementia across the spectrum of mild cognitive impairment. Arch Gen Psychiatry. 2007;64(12):1443-1450. 18056553 10.1001/archpsyc.64.12.1443 PMC2581771

[14] Aisen PS, Andrieu S, Sampaio C, et al. Report of the task force on designing clinical trials in early (predementia) AD. Neurology. 2011;76(3):280-286. 21178097 10.1212/WNL.0b013e318207b1b9 PMC3034393

[15] Blennow K, Hampel H, Weiner M, Zetterberg H. Cerebrospinal fluid and plasma biomarkers in Alzheimer disease. Nat Rev Neurol. 2010;6(3):131-144. 20157306 10.1038/nrneurol.2010.4

[16] Palmqvist S, Zetterberg H, Blennow K, et al. Accuracy of brain amyloid detection in clinical practice using cerebrospinal fluid beta-amyloid 42: a cross-validation study against amyloid positron emission tomography. JAMA Neurol. 2014;71(10):1282-1289. 25155658 10.1001/jamaneurol.2014.1358

[17] Bertens D, Tijms BM, Scheltens P, Teunissen CE, Visser PJ. Unbiased estimates of cerebrospinal fluid beta-amyloid 1-42 cutoffs in a large memory clinic population. Alzheimers Res Ther. 2017;9(1):8. 28193256 10.1186/s13195-016-0233-7 PMC5307885

[18] Benaglia T, Chauveau D, Hunter DR, Young DS. Mixtools: an R Package for analyzing mixture models. J Stat Softw. 2009;32(6):1-29.

[19] Palmqvist S, Minthon L, Wattmo C, Londos E, Hansson O. A Quick Test of cognitive speed is sensitive in detecting early treatment response in Alzheimer's disease. Alzheimers Res Ther. 2010;2(5):29. 20950460 10.1186/alzrt53 PMC2983438

[20] Fritz CO, Morris PE, Richler JJ. Effect size estimates: current use, calculations, and interpretation. J Exp Psychol Gen. 2012;141(1):2-18. 21823805 10.1037/a0024338

[21] Yost KJ, Cella D, Chawla A, et al. Minimally important differences were estimated for the Functional Assessment of Cancer Therapy-Colorectal (FACT-C) instrument using a combination of distribution- and anchor-based approaches. J Clin Epidemiol. 2005;58(12):1241-1251. 16291468 10.1016/j.jclinepi.2005.07.008

[22] Middel B, van Sonderen E. Statistical significant change versus relevant or important change in (quasi) experimental design: some conceptual and methodological problems in estimating magnitude of intervention-related change in health services research. Int J Integr Care. 2002;2:e15. 16896390 10.5334/ijic.65 PMC1480399

[23] Cornett KMD, Menezes MP, Bray P, et al; CMTPedS Study Group. Refining clinical trial inclusion criteria to optimize the standardized response mean of the CMTPedS. Ann Clin Transl Neurol. 2020;7(9):1713-1715. 32762141 10.1002/acn3.51145 PMC7480902

[24] Wolinsky FD, Unverzagt FW, Smith DM, Jones R, Stoddard A, Tennstedt SL. The ACTIVE cognitive training trial and health-related quality of life: protection that lasts for 5 years. J Gerontol A Biol Sci Med Sci. 2006;61(12):1324-1329. 17234829 10.1093/gerona/61.12.1324

[25] Rossetti HC, Munro Cullum C, Hynan LS, Lacritz LH. The CERAD neuropsychologic battery total score and the progression of Alzheimer disease. Alzheimer Dis Assoc Disord. 2010;24(2):138-142. 20505431 10.1097/WAD.0b013e3181b76415 PMC2920638

[26] Unger JM, van Belle G, Heyman A; Consortium to Establish a Registry for Alzheimer's Disease. Cross-sectional versus longitudinal estimates of cognitive change in nondemented older people: a CERAD study. J Am Geriatr Soc. 1999;47(5):559-563. 10323649 10.1111/j.1532-5415.1999.tb02570.x

[27] Malec JF, Ketchum JM. A standard method for determining the minimal clinically important difference for rehabilitation measures. Arch Phys Med Rehabil. 2020;101(6):1090-1094. 31953077 10.1016/j.apmr.2019.12.008

[28] Leidy NK, Wyrwich KW. Bridging the gap: using triangulation methodology to estimate minimal clinically important differences (MCIDs). COPD. 2005;2(1):157-165. 17136977 10.1081/copd-200050508

[29] Alma H, de Jong C, Tsiligianni I, Sanderman R, Kocks J, van der Molen T. Clinically relevant differences in COPD health status: systematic review and triangulation. Eur Respir J. 2018;52(3):1800412. 30139774 10.1183/13993003.00412-2018

[30] Burnham KP, Anderson DR. Multimodel inference: understanding AIC and BIC in model selection. Sociol Methods Res. 2004;33(2):261-304.

[31] Olofsen E, Dahan A. Using Akaike's information theoretic criterion in mixed-effects modeling of pharmacokinetic data: a simulation study. F1000Research. 2013;2:71. 26673949 10.12688/f1000research.2-71.v1 PMC4670010

[32] Andrews JS, Desai U, Kirson NY, Zichlin ML, Ball DE, Matthews BR. Disease severity and minimal clinically important differences in clinical outcome assessments for Alzheimer's disease clinical trials. Alzheimers Dement (NY). 2019;5:354-363. 10.1016/j.trci.2019.06.005 PMC6690415 31417957

[33] Howard R, Phillips P, Johnson T, et al. Determining the minimum clinically important differences for outcomes in the DOMINO trial. Int J Geriatr Psychiatry. 2011;26(8):812-817. 20848576 10.1002/gps.2607

[34] Watt JA, Veroniki AA, Tricco AC, Straus SE. Using a distribution-based approach and systematic review methods to derive minimum clinically important differences. BMC Med Res Methodol. 2021;21(1):41. 33637039 10.1186/s12874-021-01228-7 PMC7912575

[35] Burback D, Molnar FJ, St John P, Man-Son-Hing M. Key methodological features of randomized controlled trials of Alzheimer's disease therapy. Minimal clinically important difference, sample size and trial duration. Dement Geriatr Cogn Disord. 1999;10(6):534-540. 10559571 10.1159/000017201

[36] Schrag A, Schott JM. Alzheimer's Disease Neuroimaging Initiative. What is the clinically relevant change on the ADAS-Cog?. J Neurol Neurosurg Psychiatry. 2012;83(2):171-173. 22019547 10.1136/jnnp-2011-300881

[37] Wada-Isoe K, Kikuchi T, Umeda-Kameyama Y, Mori T, Akishita M, Nakamura Y. ABC Dementia Scale Research Group. Global clinical dementia rating score of 0.5 may not be an accurate criterion to identify individuals with mild cognitive impairment. J Alzheimers Dis Rep. 2019;3(1):233-239. 31754655 10.3233/ADR-190126 PMC6839533

[38] Coley N, Andrieu S, Jaros M, Weiner M, Cedarbaum J, Vellas B. Suitability of the clinical dementia rating-sum of boxes as a single primary endpoint for Alzheimer's disease trials. Alzheimers Dement. 2011;7(6):602.e2-610.e2. 21745761 10.1016/j.jalz.2011.01.005

[39] Cedarbaum JM, Jaros M, Hernandez C, et al. Alzheimer's Disease Neuroimaging Initiative. Rationale for use of the clinical dementia rating sum of boxes as a primary outcome measure for Alzheimer's disease clinical trials. Alzheimers Dement. 2013;9(1 suppl):S45-S55. 22658286 10.1016/j.jalz.2011.11.002

[40] Revicki D, Hays RD, Cella D, Sloan J. Recommended methods for determining responsiveness and minimally important differences for patient-reported outcomes. J Clin Epidemiol. 2008;61(2):102-109. 18177782 10.1016/j.jclinepi.2007.03.012

[41] Insel PS, Weiner M, Mackin RS, et al. Determining clinically meaningful decline in preclinical Alzheimer disease. Neurology. 2019;93(4):e322-e333. 31289148 10.1212/WNL.0000000000007831 PMC6669933

[42] Papp KV, Rentz DM, Orlovsky I, Sperling RA, Mormino EC. Optimizing the preclinical Alzheimer's cognitive composite with semantic processing: the PACC5. Alzheimers Dement (NY). 2017;3(4):668-677. 10.1016/j.trci.2017.10.004 PMC5726754 29264389

